# High Aldehyde Dehydrogenase Levels Are Detectable in the Serum of Patients with Lung Cancer and May Be Exploited as Screening Biomarkers

**DOI:** 10.1155/2019/8970645

**Published:** 2019-08-22

**Authors:** Alessandra Rossi, Minna Voigtlaender, Hans Klose, Hartmut Schlüter, Gerhard Schön, Sonja Loges, Moreno Paolini, Carsten Bokemeyer, Martin Reck, Giulio Tarro, Mascha Binder

**Affiliations:** ^1^Department of Oncology, Hematology and Bone Marrow Tansplantation with Section Pneumology, Hubertus University Medical Center Hamburg-Eppendorf, Hubertus Wald Tumorzentrum (University Cancer Center Hamburg), University Medical Center Hamburg, 25 Martinistrasse Str., Hamburg, Germany; ^2^Department of Pharmacy and Biotechnologies, Alma Mater Studiorum, University of Bologna, 44 Irnerio Str., Bologna, Italy; ^3^Department of Clinical Chemistry, Core Facility Mass Spectrometric Proteomics, University Medical Center Hamburg, 25 Martinistrasse Str., Hamburg, Germany; ^4^Department of Medical Biometry and Epidemiology, University Medical Center Hamburg-Eppendorf, 25 Martinistrasse Str., Hamburg, Germany; ^5^Department of Thoracic Oncology, LungenClinic Grosshansdorf GmbH, Airway Research Center North (ARCN), Member of the German Centre for Lung Research (DZL), 80 Wöhrendamm Str., Grosshansdorf, Germany; ^6^T. & L. de Beaumont Bonelli Foundation, Naples, 286 Posillipo Str., Naples, Italy; ^7^Committee on Biotechnologies and Virus Sphere, World Academy of Biomedical Technologies, UNESCO, Paris, France; ^8^Department of Inner Medicine IV, Hematology and Oncology, University Hospital Halle, Ernst-Grube-Str. 40, D–06120 Halle (Saale), Germany

## Abstract

**Objectives:**

Since early detection improves overall survival in lung cancer, identification of screening biomarkers for patients at risk represents an area of intense investigation. Tumor liberated protein (TLP) has been previously described as a tumor-associated antigen (complex) present in the sera from lung cancer patients. Here, we set out to identify the nature of TLP to develop this as a potential biomarker for lung cancer screening.

**Materials and Methods:**

Beginning from the peptide epitope RTNKEASI previously identified from the TLP complex, we produced a rabbit anti-RTNKEASI serum and evaluated it in the lung cancer cell line A549 by means of immunoblot and peptide completion assay (PCA). The TLP sequence identification was conducted by mass spectrometry. The detected protein was, then, analyzed in patients with non-small cell lung cancer (NSCLC) and benign lung pathologies and healthy donors, by ELISA.

**Results:**

The anti-RTNKEASI antiserum detected and immunoprecipitated a 55 kDa protein band in the lysate of A549 cells identified as aldehyde dehydrogenase isoform 1A1, revealing the molecular nature of at least one component of the previously described TLP complex. Next, we screened blood samples from a non-tumor cohort of 26 patients and 45 NSCLC patients with different disease stages for the presence of ALDH1A1 and global ALDH. This analysis indicated that serum positivity was highly restricted to patients with NSCLC (ALDH *p* < 0.001; ALDH1A1 *p*=0.028). Interestingly, the global ALDH test resulted positive in more NSCLC samples compared to the ALDH1A1 test, suggesting that other ALDH isoforms might add to the sensitivity of the assay.

**Conclusion:**

Our data indicate that ALDH levels are elevated in the sera of NSCLC patients, even with early stage disease, and may thus be evaluated as part of a marker panel for non-invasive detection of NSCLC.

## 1. Introduction

Despite various treatment approaches such as surgery, radiotherapy, and chemotherapy, lung cancer remains the most common cancer-related cause of death in the world with a 15% 5-year survival rate of about [1–3]. Non-small cell lung cancer (NSCLC) represents the most frequent histology and accounts for 80–85% of newly diagnosed cases. The standard of care for functionally operable early-stage and resectable stage IIIA NSCLC is surgery which possesses a potential for cure. Nevertheless, only 20% of NSCLC are diagnosed at early stage and can be resectable; thus, early detection strategies remain an unmet clinical need [4–6].

So far, numerous studies investigated mainly the potential effects of chest X-rays and low-dose helical computed tomography (CT) for imaging alongside with sputum cytology on lung cancer detection. Although these studies showed encouraging results about stage distribution in favor of earlier stage disease, better surgical resection of the tumors, and also an increased survival rate, an improvement on overall mortality could not be determined [7–16]. Serological markers such as Carcinoembryonic antigen (CEA), neuron-specific enolase (NSE), and Cyfra 21-1 are serological markers used for the monitoring of treatment response in lung cancer patients, but their application as screening biomarkers is still a challenging question [17–19]. Theoretically, an ideal biomarker should be 100% sensitive and specific, a goal that is almost never accomplished. One strategy potentially increasing both parameters is to putting different biomarkers together into a screening marker panel. This approach, along with other noninvasive methods, may allow for further improvement of NSCLC screening [20].

In 1983, a tumor-associated antigen was isolated from NSCLC and named tumor liberated protein (TLP) by Tarro et al. and immunohistochemically localized in small and large granules into the cytoplasm [4, 21–25]. Given that it was also detected in the lumen of atypical glands and in the bronchial secretions of some specimens, TLP could be considered a secretory product of cancer cells. It has been shown that when TLP is isolated and purified from a patient's tumor and reintroduced into the body, it boosts an immune response in the host [4]. Starting from the partial sequence analysis of this protein, corresponding antigenic peptides have been synthetized and used to generate antisera in rabbits. Among the four TLP-derived peptides identified by Tarro, anti-RTNKEASI rabbit sera reacted specifically with NSCLC tumor extracts and sera from lung cancer patients [26]. TLP was detected in the sera of NSCLC patients above all at the early stage of disease.

In this study, we show that TLP (or a component of this putative complex) corresponds to aldehyde dehydrogenase (ALDH) isoform 1A1 (ALDH1A1). ALDHs are a broad family of intracellular enzymes that are involved in cellular detoxification, differentiation, and drug resistance processes by means of the conversion of exogenous and endogenous aldehydes to carboxylic acids [4, 6, 27–30]. Numerous studies have investigated the biological role of ALDH in cancers including breast cancer, colon cancer, head and neck, papillary thyroid carcinoma, and mainly lung cancer, where they have given supportive evidence for the correlation between ALDH activity and lung cancer stem cells [4, 31–37]. Moreover, Cao et al. showed that ALDH1A1 levels were elevated in the sera of NSCLC patients. The combination of serum ALDH1A1 and CEA significantly increased the screening sensitivity of single CEA test [38]. Here, we show that ALDH isoforms other than 1A1 may be released into the blood of NSCLC patients, and thus, screening sensitivity may be even more improved by employing an isoform-unspecific global ALDH assay without apparently lowering specificity. Diagnostic sensitivity and specificity will, however, have to be prospectively validated in larger cohorts of patients with early-stage NSCLC and in healthy subjects at risk for NSCLC as well as in other cancer patient cohorts.

## 2. Materials and Methods

### 2.1. Patient Characteristics and Materials

Blood samples from 45 newly diagnosed NSCLC patients, 17 patients with benign lung pathologies, and 9 healthy donors were collected during routine diagnostic workup. The work has been carried out in accordance with the declaration of Helsinki. All patients consented to the use of their biological material for this investigation as approved by the Landesärztekammer Hamburg (ethics committee) (project number PV4382). Patients with lung cancer stages I-IIIA were considered as early-stage disease, as previously published [30]. Clinical characteristics (type of lung cancer, patient age, sex, smoking, tumor histology, tumor stage, and secondary diagnosis) of this cohort are displayed in Table 1.

### 2.2. Antigen Synthesis and Antibody Production

The production of two different rabbit polyclonal anti-RTNKEASI immune sera and the synthesis of TLP-derived peptide RTNKEASI [26] were both conducted at Rockland Immunochemicals Inc. (Gilbertsville, PA, USA) and at BioGenes GmbH (Berlin, Germany). We further purified the anti-RTNKEASI immune serum produced at Rockland by means of chromatography against the RTNKEASI peptide.

### 2.3. Cell Culture

The human cell lines MRC-5, A549, Hela, CA46, HL60, PC3, and MCF-7 were obtained from American Type Culture Collection (ATCC; Manassas, VA, USA). Cell lines were cultured at 37°C in a humidified atmosphere of 5% CO_2_ in air under the following conditions: CA46 cell line in Glutamax RPMI 1640; PC3 cell line in Glutamax HAM's F-12; MRC-5, HL60, MCF-7, A549, and Hela cell lines in Glutamax Dulbecco's Modified Eagle's Medium. All the media (Gibco, Life Technologies) were supplemented with 10% fetal bovine serum (Biochrom GmbH, Berlin, Germany) [39].

### 2.4. Western Blot

We prepared cell pellets and lysates from MCF-7, CA46, HL60, PC3 MRC-5, A549, and Hela cell lines as described previously [39, 40].

Cell culture supernatant from A549 cells (5 × 10^6^) was treated on ice with 10% trichloroacetic acid (TCA) and then incubated with ice-cold 90% acetone at −20°C. Subsequently, the sample was centrifuged and the pellet dried at 65°C for 30 minutes.

The isolated proteins were mixed in Laemmli sample buffer (20% glycerol, 4% sodium dodecyl sulfate (SDS), 100 mM Tris-HCl, 200 mM dithiothreitol (DTT), and 0.01% bromophenol blue) and boiled before loading into SDS-PAGE gel. Finally, the proteins were blotted onto membranes and blocked in nonfat dry milk 8% + PBS + 0.1% Tween-20 before hybridization with the anti-RTNKEASI serum diluted 1 : 1000 in PBS + 0.1% Tween-20 + BSA 5% buffer or *β*-actin 1 : 20000 (Biolegend, San Diego, CA), overnight at 4°C. After primary antibodies, the membranes were probed with a secondary antibody (1 : 30000 in PBS + 0.1% Tween-20 + BSA 5%), and then, proteins were visualized by enhanced chemiluminescence (GE Healthcare, Little Chalfont, UK).

### 2.5. PCA

The anti-RTNKEASI serum (1 : 1000) was subjected to preincubation with peptide RTNKEASI (500-fold molar excess) or KDSGNEQTFLPP as a control peptide, before hybridization with blotted A549 sample for 2 hours, according to Rockland procedure. The membranes were probed with a secondary antibody, and immunoreactive bands were detected by enhanced chemiluminescence.

### 2.6. 2D Polyacrylamide Gel Electrophoresis

Protein isolation from cell lines was performed as described previously [41]. For the first dimension isoelectric focusing, we used 7 cm immobilized pH gradient (IPG) dry strips with a linear pH 4–7 gradient (GE Healthcare). Solubilized proteins (50 *μ*g) were put onto the strips, rehydrated, and incubated over night [41]. Proteins were resolved by the PROTEAN IEF system (Bio-Rad) (voltage gradient at 20°C with a current limit of 50 *μ*A) under the following conditions: 4 h at 250 V, 8000 V linear gradient to 15000 V·h, and rapid 8000 V to 75000 V h, for a total of 90 kV·h [41]. After equilibration in buffer 1 (130 mM DTT, 6 M urea, 20% glycerol, 0.05 M Tris-HCl, 2% SDS) and buffer 2 containing iodoacetamide in place of DTT, the first dimension strips were positioned to the upper part of 8% acrylamide (Applichem GmbH, Darmstadt, Germany), for performing the second dimension [41]. The separated proteins were stained by silver or blotted onto the membrane and probed with the pre-serum or anti-RTNKEASI serum.

### 2.7. Protein Identification by LC-MS/MS

The gel spots were treated with DTT (10 mM, 56°C, 30 min) and with iodoacetamide (55 mM, at room temperature, 20 min, in the dark). Subsequently, the proteins were digested by 13 ng/*μ*l trypsin (sequencing grade modified trypsin, Promega) in 50 mM ammonium bicarbonate, 37°C, 16 h. Tryptic digested peptides were extracted in 65% acetonitrile (ACN)/5% formic acid (FA), vacuum-dried, and dissolved in 15 *μ*L 0.1% FA. Tryptic digested peptides were separated on an UltiMate 3000 Rapid Separation liquid chromatography (RSLC) system (Dionex, Thermo Scientific, Bremen, Germany) coupled online via electrospray-ionization (ESI) to an Orbitrap Fusion Tribrid mass spectrometer (Thermo Scientific, Bremen, Germany). The samples were loaded with a flow rate of 5 *μ*l/min on a trapping column (Acclaim PepMap *μ*-precolumn, C18, 300 *μ*m × 5 mm, 5 *μ*m, 100 Ǻ, Thermo Scientific, Bremen, Germany; nanoACQUITY-UPLC Symmetry C18 trap column, 180 *μ*m × 20 mm, 5 *μ*m, 100 Ǻ; buffer A: 0.1% FA in HPLC-H_2_O; buffer B: 0.1% FA in ACN) with 2% buffer B. The loaded samples on the trapping column were washed firstly for 5 min with 2% buffer B (5 *μ*l/min) and subsequently the peptides were eluted (200 nl/min) onto the separation column. Tryptic digested peptides were separated on a reversed-phase C18 column (Acclaim PepMap 100, 75 *μ*m × 25 mm, 2 *μ*m, 100 Å; Thermo Scientific, Bremen, Germany; nanoACQUITY-UPLC column, BEH 130 C18, Waters; 75 *μ*m × 250 mm, 1.7 *μ*m, 100 Ǻ) with a flow rate 200 nl/*μ*m with a binary buffer system of solvent A (0.1% FA in HPLC-H_2_O) and solvent B (0.1% FA in ACN). The peptides were eluted with a gradient of 2–30% Solvent B in 30 min. MS analysis was performed in the positive ion mode and was programmed to acquire by data-dependent mode (DDA). The full scans were acquired in the Orbitrap mass analyzer of Fusion with a resolution of 120,000 FWHM at *m*/*z* 200 on the MS level over a *m*/*z* range from 400 to 1500 (maximum injection time: 50 ms and automatic gain control target: 4*e*5). The fragmentation was carried out with an intensity threshold of 1*e*4, and the fragmented ions were accumulated in the linear ion trap in the rapid mode. Only precursors with charge states between +2 and +5 and the most intense precursors were selected for fragmentation. The top intense ions were isolated to a target value of 1*e*4 with a maximum injection time of 50–150 ms. The raw data were processed with Proteome Discoverer, v1.4.1.14 (Thermo Scientific). Protein identification was performed by using UniProt FASTA database.

### 2.8. ALDH and ALDH1A1 ELISA Assay

Serum samples from patients with NSCLC and benign lung pathologies and healthy donors were collected and stored at −80°C. The sera were subsequently assayed according to the manual instructions of the global ALDH ELISA kit with a monoclonal antibody specific for different ALDH isoforms (Bluegene, Biotech, Shanghai, China) and the ALDH1A1 ELISA kit with a monoclonal antibody specific for the isoform 1A1 (Cloud-Clone Corp. Houston, TX, USA). Three independent sera patients' samples are analyzed. Data are expressed as means ± SD of 3 independent experiments.

### 2.9. Statistics

Data were presented as means ± standard deviation (SD). We used backward stepwise linear-regression modelling to analyze differences in the ALDH- and ALDH1A1 serum levels, respectively, between patients with and without NSCLC under consideration of age, sex, and smoking (in pack-years). Correlation coefficients were according to the method of Pearson. All analyses were carried out using IBM® SPSS® version 22 or GraphPad Prism™ version 5 software. A *p* value of <0.05 was considered statistically significant.

## 3. Results

### 3.1. Peptide RTNKEASI Mimics a 55 kDa Protein Highly Expressed in Lung Cancer Cell Line A549

**Figure 1:**
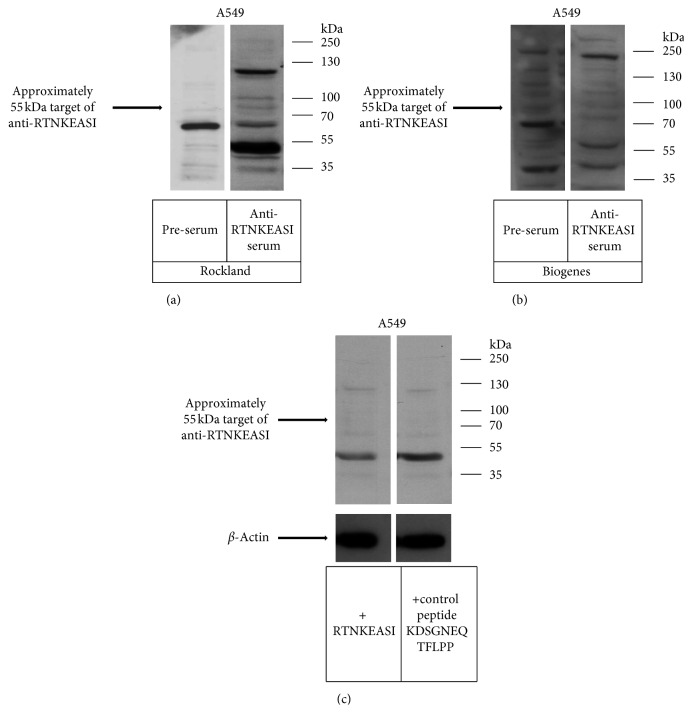
Western blot analysis of the polyclonal anti-RTNKEASI serum in A549 cell line. SDS gel was loaded with protein extract from non-small cell lung cancer A549 cell line followed by electrophoresis and immunoblotting. The blots were incubated with the preimmune serum and the polyclonal anti-RTNKEASI serum produced in rabbits from Rockland (a) or BioGenes (b). The 55 kDa target of anti-RTNKEASI appears only in sera of animals after immunization, and it is absent in the presera from the same rabbit. These results were confirmed with both Rockland and BioGenes antibodies. A PCA was performed by incubating the corresponding blots with the polyclonal anti-RTNKEASI serum from Rockland, pretreated with or without the peptide RTNKEASI (c) and also *β*-actin, as endogenous control. The intensity of the band approximately at 55 kDa detected by the secondary anti-rabbit horseradish peroxidase-conjugated was reduced after the preincubation of the antibody with the peptide RTNKEASI.

To identify the complete protein sequence or major component of TLP, we produced two polyclonal antibody sera (BioGenes GmbH, Berlin, Germany; Rockland Immunochemicals, PA, USA) by using the peptide epitope mimic RTNKEASI derived from TLP as previously published [25]. Compared to the preimmune sera, both anti-RTNKEASI sera detected one differential protein band in the NSCLC cell line A549 at 55 kDa (Figures 1(a) and 1(b)).

Based on these results, we performed a peptide competition assay (PCA) with limiting concentrations of detection antiserum and an excess of blocking RTNKEASI or control peptide. A partial extinction of the 55 kDa protein band in the presence of the specifically blocking RTNKEASI peptide compared to the control peptide confirmed that the 55 kDa band was specifically recognized by RTNKEASI-directed antibodies from the antiserum (Figure 1(c)).

**Figure 2:**
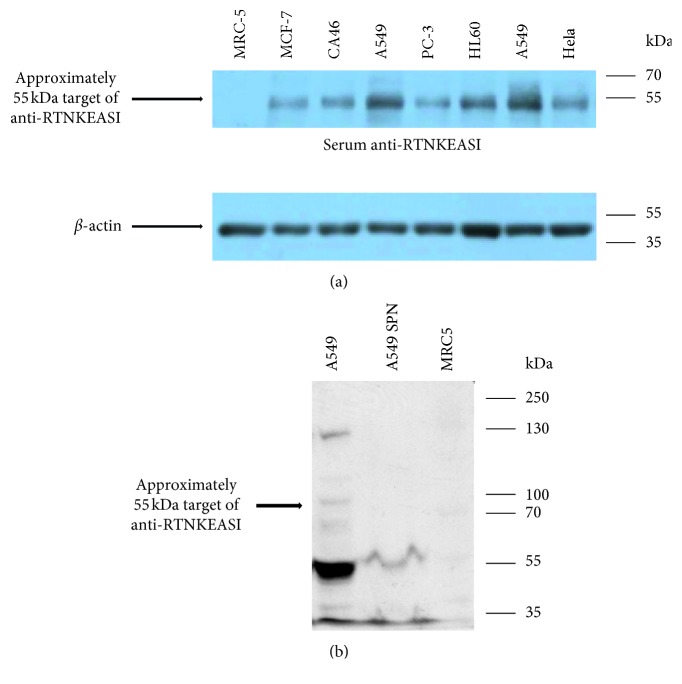
Western blot analysis for the polyclonal anti-RTNKEASI serum in cancer cell lines and cellular supernatant. (a) Protein extracts from non-small cell lung cancer A549, normal lung tissue MRC-5, Burkitt lymphoma CA46, leukemia HL60, breast cancer MCF-7, cervical carcinoma Hela, and prostate cancer PC-3 cell lines were loaded into SDS gel and subjected to electrophoresis and immunoblotting with the polyclonal anti-RTNKEASI serum from Rockland. High 55 kDa protein levels were identified in A549 cells and a lower amount in Hela, PC-3, CA46 and HL-60 cell lines whereas no protein levels were observed in MCF-7 cell line from breast cancer and in MRC-5 from normal lung tissue. An anti-ß-actin antibody was used for a loading control. (b) Protein extracts from cellular supernatant of non-small cell lung cancer A549 and cell lysate from A549 and MRC-5 cells were loaded into SDS gel and subjected to electrophoresis and immunoblotting with the polyclonal anti-RTNKEASI serum from Rockland. A specific band at 55 kDa was detected in the cellular supernatant sample with respect to cell lysate from A549; MRC-5 was used as negative control.

In order to analyze specificity of the 55 kDa protein for lung cancer, we subjected protein lysates from a variety of tumor cell lines and normal lung tissue to western blot analysis with the anti-RTNKEASI serum. Our western blot results showed very high levels of this 55 kDa protein in the lung cancer cell line A549, whereas all other cell lines showed either no or faint bands at 55 kDa (Figure 2(a)).

We also analyzed the supernatant from A549 cell line to verify cell ability to secrete ALDH outside. Our western blot showed a specific band at 55 kDa with a considerable amount of this protein corresponding to ALDH, as confirmed by mass spectrometry analysis (Figure 2(b) and Table 2).

### 3.2. Mass Spectrometric Identification of ALDH1A1 as Parental Antigen Mimicked by Peptide RTNKEASI

**Figure 3:**
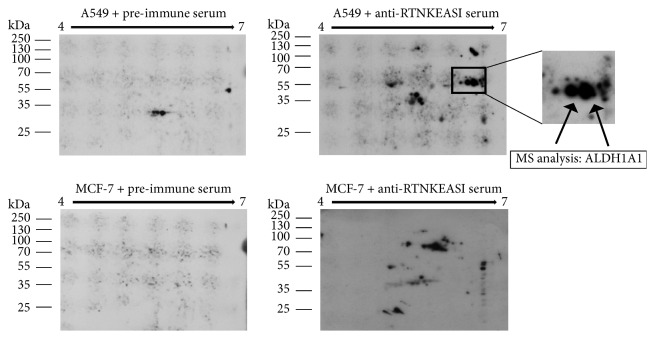
2D gel electrophoresis and western blot analysis with cell lysates from A549 and MCF-7 cell lines using the polyclonal anti-RTNKEASI serum and preimmune serum. Protein extract from A549 and MCF-7 cells were loaded in 2D gel followed by electrophoresis and transferred to PVDF for immunoblotting with the polyclonal anti-RTNKEASI serum or preserum. A rectangle marks the location of the ALDHs detected with the polyclonal anti-RTNKEASI serum in A549 cells; the reactive spots indicated with arrows were absent in MCF-7 cells and in the blot incubated with the preserum. These protein spots at 55 kDa, excited and subjected to MS analysis, correspond to ALDH1A1.

In order to identify the 55 kDa protein by mass spectrometry (MS), we conducted a two-dimensional (2D) gel electrophoresis of protein extracts from A549 and MCF-7 (negative control) followed by western blotting and detection with the anti-RTNKEASI serum (Figure 3). We observed two neighboring spots at the same molecular weight of 55 kDa but with slightly different isoelectric points, 6.54 and 6.73 respectively, which exhibited reactivity with the anti-RTNKEASI serum. These spots were neither detected by the preimmune serum nor by the anti-RTNKEASI serum in the control cell lysate (Figure 3). These two spots were matched to and excised from 2D protein gels followed by MS. Our results showed that the 55 kDa protein corresponds to ALDH isoform 1A1 which is highly expressed in different tumors including NSCLC. This result was also confirmed by MS of one dimensionally (1D) separated protein extracts from A549 cells, A549 cell culture supernatant, and immunprecipitations with anti-RTNKEASI serum from the A549 lysate (Table 2). Sequence alignment of ALDH1A1 with the peptide RTNKEASI revealed no linear matches, suggesting that this peptide only structurally mimics ALDH1A1.

### 3.3. Detection of ALDH1A1 and Global ALDH in Sera of Patients with Lung Cancer and Benign Lung Pathologies

**Figure 4:**
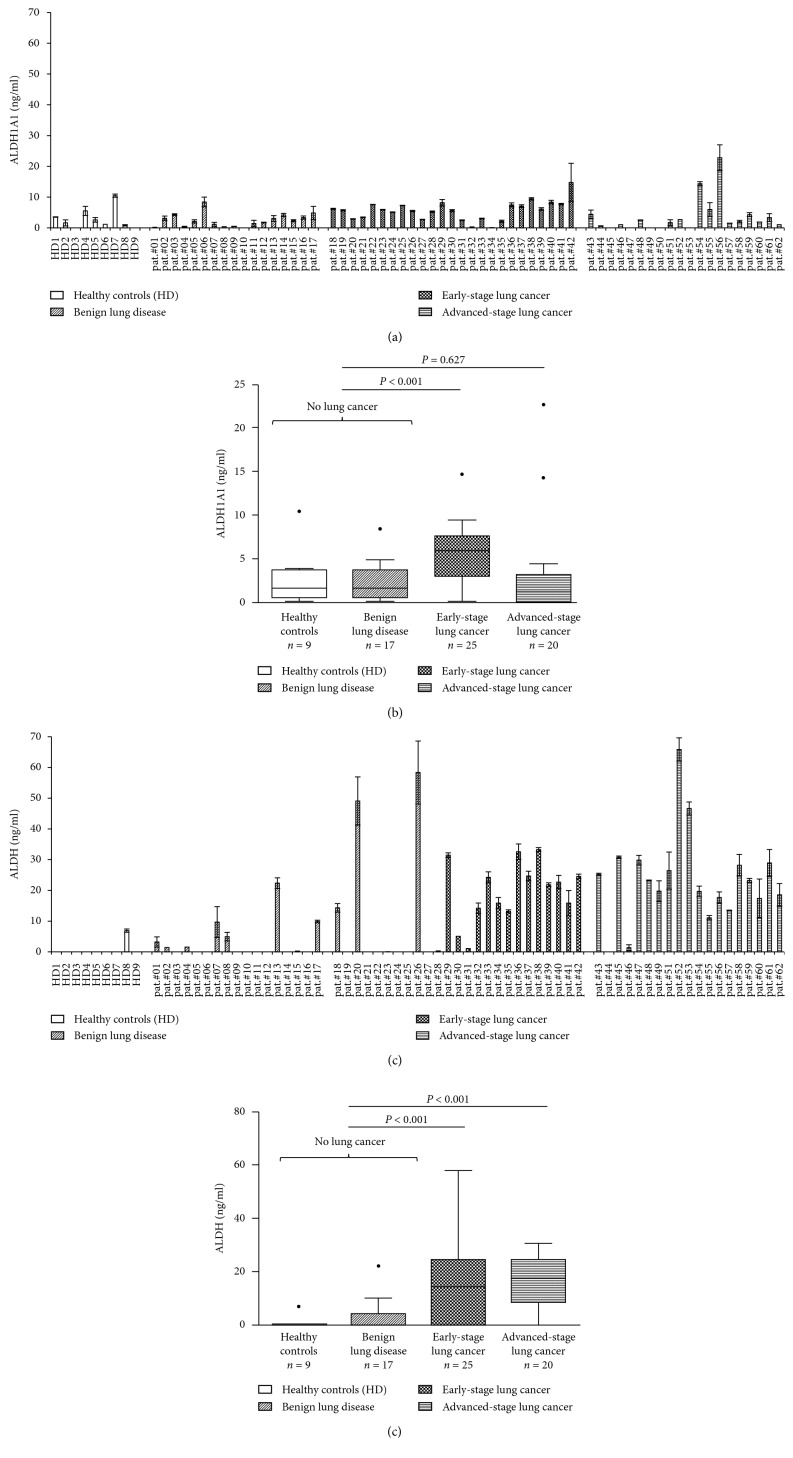
ELISA assay for sera (a, b) ALDH1A1 and (c, d) ALDHs. Sera samples from patients with NSCLC and benign lung pathologies and healthy donors were collected and subjected to an enzyme-linked immunosorbent assay with a monoclonal antibody recognizing specifically the isoform ALDH1A1 and all ALDH isoforms, respectively. (a) and (c) show individual data points, expressed as means ± SD of 3 independent experiments, while (b) and (d) show summary statistics. Boxes and whiskers represent median and (interquartile) range. A backward stepwise linear-regression modelling was used to analyze differences in ALDH1A1 and ALDH concentrations between patients with and without lung cancer after precluding age, sex, and smoking (in pack-years) as nonsignificant independent covariables.

To explore the utility of serum ALDH1A1 as potential biomarker for lung cancer, we tested ALDH1A1 protein levels in sera from 25 NSCLC patients with early-stage disease, 20 NSCLC patients with advanced stage disease, 17 patients with nonneoplastic pulmonary diseases and 9 healthy donors. The baseline characteristics of this cohort are shown in Table 1. We compared ALDH serum levels in NSCLC patients with benign lung pathologies and healthy donors groups as negative controls to set the threshold of background and normalize the measurements, according to previous studies [42–44]. We found ALDH1A1 serum levels above a sensitivity threshold of 10 ng/ml (reflecting the estimated background noise) in only three of 45 NSCLC patients (6.7%), while all other NSCLC patients showed ALDH1A1 serum levels comparable with the control cohorts (Figures 4(a) and 4(b)). However, after precluding age, sex, and smoking (in pack-years) as nonsignificant parameters associated with the concentration of ALDH1A1 in the backward stepwise linear-regression analysis, overall, ALDH1A1 serum levels differed significantly between the lung cancer and the no-lung cancer patient groups. The mean difference in the ALDH1A1 concentration between no lung cancer and lung cancer is 2.10 ng/ml, 95% CI: 0.23 ng/ml to 3.98 ng/ml; *p*=0.028.

Since the MS analysis had also revealed matches with other ALDH isoforms (such as ALDH3A1), we hypothesized that these might add to the sensitivity of the assay. To this end, we performed further ELISA testings using a global ALDH assay without isoform specificity (Figures 4(c) and 4(d)). Remarkably, 33 out of 45 serum samples (73.3%) showed positivity for global serum ALDH (>10 ng/ml). Only one patient from the cohort of patients with benign lung diseases showed ALDH serum positivity above this threshold, and all healthy donors were serum ALDH-negative. Interestingly, of 25 patients with early-stage NSCLC, 15 patients (60%) showed elevated ALDH levels. Overall, serum ALDH levels were significantly elevated in the cohort of patients with NSCLC compared to patients without lung cancer. The mean difference in the ALDH concentration between no lung cancer and lung cancer is 13.90 ng/ml, 95% CI: 8.45 ng/ml to 19.35 ng/ml; *p* < 0.001. All the statistical analysis values are reported in Supplementary Materials ([Supplementary-material supplementary-material-1] and [Supplementary-material supplementary-material-1]).

According to the method of Pearson, neither ALDH1A1 nor ALDH concentration correlated significantly with the UICC stage (*p*=0.113 and *p*=0.359, respectively).

## 4. Discussion

Worldwide, NSCL patients have the highest mortality between patients with solid tumors, and their prognosis is tightly stage-correlated. Nevertheless, conventional methods for the diagnosis of NSCLC have high costs and produce potentially false-positive outcomes. Thus, the discovery of highly sensitive, specific, noninvasive, and cost-effective lung cancer biomarkers to use in association with conventional approaches may increase the sensitivity of NSCLC screening [4, 6, 45–47].

In this study, we show that the previously described TLP corresponds to ALDH1A1 and potentially other ALDH isoforms, which are highly expressed in NSCLC tissues [28, 33, 35–38, 46–49]. Since the peptide RTNKEASI did not linearly match ALDH isoform amino acid sequences, we concluded that it may structurally mimic these ALDH isoforms.

In our blood-based ELISA assays, we show that ALDH and, in accordance with Cao et al. [38], ALDH1A1 protein levels are statistically higher in patients with NSCLC compared to our nontumor cohort. However, only a small percentage of NSCLC patients (6.7%) display high ALDH1A1 serum levels, whereas sensitivity of the global ALDH test seems encouraging (73.3%). This suggested that other ALDH isoforms are also released in the sera of NSCLC patients potentially adding to the sensitivity of this global assay. This finding is in accordance with previous studies showing that several ALDH isoforms are involved in NSCLC [38]. ALDH3A1 is highly expressed in two types of NSCLC, adenocarcinoma and squamous cell carcinoma [48, 49]. ALDH3B1 expression was also found to be upregulated in a high percentage of human tumors, particularly in lung cancer [50]. Given the limited sample sizes of our cohorts, predictions on sensitivity and specificity of this marker are unreliable. However, it seems that the global ALDH test may not significantly decrease specificity as compared to the ALDH1A1-specific test.

ALDH1A1 and ALDH3A1 expression levels in normal pneumocytes are significantly higher in tobacco smokers versus nonsmokers [48]. Although we did not identify any significant association between ALDH levels and smoking status across the cohorts, the higher expression levels in smokers may explain the trend towards higher ALDH levels in patients with benign lung pathologies (including a high percentage of patients with chronic obstructive pulmonary disease) compared to our nonsmoking healthy donors.

Our very small percentage of ALDH1A1-positive NSCLC patients (6.7%) clearly contrasts with previously published work by Cao et al. [38] who detected a much higher percentage of ALDH1A1-positive NSCLC patients (55%). This may be due to differences in ALDH1A1 kits, which presumably contain monoclonal antibodies with different specificity for ALDH1A1 but may also be explained in part by differences in the patient cohorts or different cut-off levels. Moreover, our results showed that some patients positive with ALDH1A1 were not with global ALDH, probably due to a potential degradation of these samples as global ALDH was performed later in time.

In conclusion, elevated ALDH serum levels can be detected in the vast majority of patients with early- and advanced-stage disease. Therefore, serum ALDH should be evaluated as part of a marker panel for noninvasive detection of early NSCLC in a larger cohort of patients at risk.

## Figures and Tables

**Table 1:** Baseline characteristics of all patients and healthy controls.

	Healthy controls (*n* = 9)	Benign lung disease (*n* = 17)	Early-stage lung cancer (*n* = 25)	Advanced-stage lung cancer (*n* = 20)
Age in years, mean ± SD	42.7 ± 18.0	53.1 ± 18.3	68.5 ± 8.2	62.3 ± 8.8
Male, no. (%)	2 (22.2)	10 (58.8)	12 (48.0)	10 (50)
Smoker, no. (%)	0	11 (64.7)	20 (80.0)	15 (88.0)^*∗*^
Smoking in pack-years, mean ± SD	0	28.4 ± 29.5	43.6 ± 30.6	47.3 ± 22.05
Type of lung cancer, no. (%)				
NSCLC			25 (100.0)	20 (100.0)
Adenocarcinoma			15 (60.0)	13 (65)
Squamous cell carcinoma			8 (32.0)	2 (10)
Large cell carcinoma			2 (8.0)	1 (5)
UICC classification, no. (%)				
IA			5 (20.0)	
IB			8 (32.0)	
IIA			6 (24.0)	
IIB			5 (20.0)	
IIIA			1 (4.0)	
IIIB				1 (5.0)
IV				15 (75.0)
Type of benign lung diseases, no. (%)				
COPD		8 (47.1)		
Cystic fibrosis		4 (23.5)		
Other benign lung diseases^*∗∗*^		5 (29.4)		

NSCLC: non-small cell lung cancer; COPD: chronic obstructive pulmonary disease. ^*∗*^Smoking status of 3 patients unknown. ^*∗∗*^Other benign lung diseases comprise precapillary pulmonary hypertension (*n* = 2), interstitial lung disease (*n* = 1), sarcoidosis (*n* = 1), and mucoid impaction (*n* = 1).

**Table 2:** Mass spectrometric identification of aldehyde dehydrogenase as 55 kDa target of the anti-RTNKEASI serum.

Gene symbol	Protein description	Unique peptides	Score	MW (kDa)
55 kDa band from A549 cell lysate excised from 1D gel				
ALDH1A1	Retinal dehydrogenase	26	687	55.4
ALDH3A1	Aldehyde dehydrogenase, dimeric NADP-preferring	15	407	50.8
UGDH	UDP-glucose 6-dehydrogenase	3	140	55.7
GSR	Glutathione reductase mitochondrial	4	134	56.8
PDIA3	Protein disulfide-isomerase A3	3	93	57.1
ALDH2	Aldehyde dehydrogenase, mitochondrial	3	83	56.9

55 kDa band from A549 supernatant excised from 1D gel				
ALDH1A1	Retinal dehydrogenase 1	29	742	55.5
G6PD	Glucose-6-phosphate 1-dehydrogenase	26	599	59.7
ALDH3A1	Aldehyde dehydrogenase, dimeric NADP-preferring	10	303	50.8

55 kDa band from A549 immunoprecipitation excised from 1D gel				
ALDH1A1	Retinal dehydrogenase 1	28	351	55.5
G6PD	Glucose-6-phosphate 1-dehydrogenase	12	223	59.7
ALDH3A1	Aldehyde dehydrogenase, dimeric NADP-preferring	3	70	50.8

55 kDa spot 1 from A549 lysate excised from 2D gel				
ALDH3A1	Aldehyde dehydrogenase, dimeric NADP-preferring	20	515	50.4
CCT2	T-complex protein 1 subunit beta	18	100.8	57.5
ALDH1A1	Retinal dehydrogenase 1	12	57.4	54.8
ALDH1B1	Aldehyde dehydrogenase X, mitochondrial	11	52	57.2

55 kDa spot 2 from A549 lysate excised from 2D gel				
ALDH3A1	Aldehyde dehydrogenase, dimeric NADP-preferring	19	494.8	50.4
ALDH1A1	Retinal dehydrogenase 1	19	120.45	54.8

MW: molecular weight.

## Data Availability

All data used to support the findings of this study are included within the article.

## Abbreviations

TLP: Tumor liberated protein
ALDH: Aldehyde dehydrogenase
NSCLC: Non-small cell lung cancer
CT: Computed tomography
CEA: Carcinoembryonic antigen
NSE: Neuron-specific enolase
ALDH1A1: Aldehyde dehydrogenase isoform 1A1
PCA: Peptide completion assay
DTT: Dithiothreitol
PVDF: Polyvinylidene difluoride
CHAPS: 3-[(3-Cholamidopropyl) dimethylammonio]-1-propanesulfonate
(ACN): Acetonitrile
FA: Formic acid
RSLC: Rapid separation liquid chromatography
DDA: Data-dependent mode
LC/MS: Liquid chromatography-mass spectrometry.

## Supplementary Materials

Supplementary Materials The first table describes the backward stepwise linear-regression analysis of the ALDH and ALDH1A1 ELISA assays, and the second table shows the mean values obtained in the related assay.
