# Supplementary material for: High Aldehyde Dehydrogenase Levels Are Detectable in the Serum of Patients with Lung Cancer and May Be Exploited as Screening Biomarkers
Source: J Oncol. 2019 Aug 22;2019:8970645. doi: 10.1155/2019/8970645 (PMC6724438; doi:10.1155/2019/8970645)
Supplement: Supplementary Materials — The first table describes the backward stepwise linear-regression analysis of the ALDH and ALDH1A1 ELISA assays, and the second table shows the mean values obtained in the related assay. [file 8970645.f1.docx]

| **Table 1 S. Backward stepwise linear-regression of ALDH and ALDH1A1 ELISA assay** | | | |
| --- | --- | --- | --- |
| **ALDH1A1** | **Mean difference (ng/ml)** | **95% CI (ng/ml)** | **P** |
| Early vs non-cancer | 3.20 ng/ml | 1.67 ng/ml - 4.73 ng/ml | <0.001 |
| Advanced vs non-cancer | 0.58 ng/ml | -1.75 ng/ml - 2.90 ng/ml | 0.627 |
| Cancer vs non-cancer | 2.10 ng/ml | 0.23 ng/ml - 3.98 ng/ml | 0.028 |
| **ALDH** |  |  |  |
| Early vs non-cancer | 13.79 ng/ml | 7.35 ng/ml - 20.24 ng/ml | <0.001 |
| Advanced vs non-cancer | 14.01 ng/ml | 9.69 ng/ml - 18.34 ng/ml | <0.001 |
| Cancer vs non-cancer | 13.90 ng/ml | 8.45 ng/ml - 19.35 ng/ml | <0.001 |

| **Table 2 S. Mean values of ALDH and ALDH1A1 ELISA assay** | |
| --- | --- |
| **ALDH1A1** | **Mean value +/- SD (ng/ml)** |
| Healthy | 2.60 ± 3.26 |
| Benign lung disease | 2.38 ± 2.16 |
| Early lung cancer | 5.66 ± 3.17 |
| Advanced lung cancer | 2.60 ± 3.26 ng/ml |
| **ALDH** |  |
| Healthy | 0.77 ± 2.30 |
| Benign lung disease | 3.14 ± 5.94 |
| Early lung cancer | 16.11 ± 16.31 |
| Advanced lung cancer | 16.36 ± 10.31 ng/ml |
